# Characterization of PAX9 variant P20L identified in a Japanese family with tooth agenesis

**DOI:** 10.1371/journal.pone.0186260

**Published:** 2017-10-12

**Authors:** Akiko Murakami, Shinji Yasuhira, Hisayo Mayama, Hiroyuki Miura, Chihaya Maesawa, Kazuro Satoh

**Affiliations:** 1 Department of Developmental Oral Health Science, School of Dentistry, Iwate Medical University, Uchimaru, Morioka, Iwate, Japan; 2 Department of Tumor Biology, Institute of Biomedical Sciences, Iwate Medical University, Nishitokuta, Yahaba-cho, Shiwa-gun, Iwate, Japan; 3 Department of Oral Medicine, School of Dentistry, Iwate Medical University, Uchimaru, Morioka, Iwate, Japan; Virginia Commonwealth University, UNITED STATES

## Abstract

Transcription factors PAX9 and MSX1 play crucial roles in the development of permanent teeth at the bud stage, and their loss-of-function variants have been associated with congenital tooth agenesis. We sequenced the coding regions of the *PAX9* and *MSX1* genes from nine patients with non-syndromic tooth agenesis, and identified a missense mutation, P20L, of *PAX9* in a single familial case involving three patients in two generations. Identical mutation was previously reported by other authors, but has not been characterized in detail. The mutation was located in a highly conserved N-terminal subdomain of the paired domain and co-segregated as a heterozygote with tooth agenesis. The patients showed defects primarily in the first and second molars, which is typical for cases attributable to PAX9 mutation. Luciferase reporter assay using the 2.3-kb promoter region of *BMP4* and electrophoretic mobility shift assay using the CD19-2(A-ins) sequence revealed that P20L substitution eliminated most of the transactivation activity and specific DNA binding activity of PAX9 under the experimental conditions we employed, while some residual activity of the mutant was evident in the former assay. The hypomorphic nature of the variant may explain the relatively mild phenotype in this case, as compared with other PAX9 pathogenic variants such as R26W.

## Introduction

Tooth agenesis is a very common congenital abnormality in humans, with a reported prevalence in the permanent dentition ranging from 1.6% to 9.6% depending on severity and the types of missing teeth [1–3]. Since the development of teeth, as well as that of other organs in the body, is controlled by coordinated action of numerous gene products, defects in any single component may potentially result in tooth absence or malformation. These mechanistic developmental networks or their components are often shared by multiple organs. Accordingly, congenital tooth agenesis may be associated with other phenotypic features in some cases (syndromic), whereas it may be isolated in others (non-syndromic). Genetic alterations responsible for syndromic or non-syndromic tooth agenesis have been described for several loci since they were first reported twenty years ago [4,5]. Most of the proteins encoded at these loci have a role in reciprocal interaction between dental epithelium and mesenchyme during tooth development [6–8]. Among them are transcription factors PAX9 and MSX1, which function in mesenchymal cells predominantly at the bud-to-cap stage and may regulate the expression of secreted signal molecules such as BMP4 [9–11]. Tooth agenesis due to loss-of-function variants of PAX9 or MSX1, which is usually non-syndromic, shows a dominant inheritance pattern in most cases, and this is ascribed to haploinsufficiency of these transcription factors. Notably, hypomorphic alleles of *PAX9* such as G6R have been reported to have a phenotype that is markedly milder than that of other pathogenic alleles [12]. Although mice with heterozygous deletion of either *Pax9* or *Msx1* do not recapitulate the phenotype observed in man, a study using homozygotes or compound heterozygotes of a series of hypomorphic *Pax9* alleles has shown that decreasing the dosage of PAX9 to a level lower than heterozygous deletion results in tooth defect, and that the phenotypic severity increases as the gene dosage decreases further [13]. These observations suggest that tooth development has a quantitative trait-like property. In humans, the dosage of PAX9 may be fine-tuned, and even slight deviation from the wild-type level may have a visible outcome, whereas in mice the minimal activity of PAX9 required for normal development may be much lower, at least for tooth phenotype. Differences in the patterns of tooth absence between PAX9 and MSX1 variants indicate the distinct functions of the gene products, whereas the phenotype of double-heterozygous mice also argues for a partially overlapping or compensatory relationship [14].

In the present study, we screened patients with moderately severe tooth agenesis for possible causative mutations in the coding region of *PAX9* and *MSX1*. We identified a missense variant, P20L, located in the paired domain of PAX9 in a single familial case. An *in vitro* experiment confirmed the detrimental effect of the mutation on PAX9 activity as a transcription factor. Thimmegowda et al. recently reported a haplotype containing both c.59C>T and c.75C>T mutations in the *PAX9* coding region in an Indian family with tooth agenesis, which would result in P20L and silent mutation at Ile25 [15,16]. However, they did not refer to the resulting amino acid change, co-segregation of the variant within the family, or the functional implications of the variant. Our study provides compelling genetic, experimental and theoretical evidence for the causal link between P20L and tooth agenesis.

## Materials & methods

### Subjects

Nine patients with tooth agenesis (three from two generations of a single family and six from sporadic cases) diagnosed at Iwate Medical University were chosen for initial mutation analysis. The patients were examined by interview and X-ray radiography prior to definitive diagnosis as non-syndromic tooth agenesis. When candidate mutations were identified, unaffected members of the same family were recruited to confirm co-segregation of the mutation with affected members. The research plan was reviewed and approved by the Institutional Review Board of the School of Dentistry at Iwate Medical University (No. 01243), and written informed consent was obtained from all subjects before blood sampling, in accordance with the Declaration of Helsinki.

### Sanger sequencing of PAX9 and MSX1

Genomic DNA was purified from samples of whole blood from the subjects using a DNA extractor WB kit (Wako, Osaka, Japan). The coding regions of *PAX9* (NM_006194.3) and *MSX1* (NM_002448.3) including several bases at intron-exon junctions were amplified with pairs of primers and KOD Plus Neo DNA polymerase (Toyobo, Osaka, Japan), and the purified PCR products were directly sequenced with a BigDye Terminator v.3.1 Cycle Sequencing Kit (Thermo Fisher Scientific, Waltham, MA, USA) and appropriate sequencing primers. The reaction was again purified with CleanSEQ (Beckman Coulter, Brea, CA, USA) following the manufacturer’s instructions and analyzed using an Applied Biosystems 3500 Genetic Analyzer (Thermo Fisher Scientific).

### Computer analysis of DNA sequences

Sequence data were compared with the corresponding reference sequences (https://www.ncbi.nlm.nih.gov/gene) and identified variants were matched with dbSNP (https://www.ncbi.nlm.nih.gov/SNP/) and ExAC (http://exac.broadinstitute.org) to eliminate common polymorphism. Candidate missense variants were analyzed with PolyPhen-2 (http://genetics.bwh.harvard.edu/pph2/index.shtml) to estimate the putative impact on protein function. Allele frequency data for known variants were retrieved from the Integrative Japanese Genome Variation Database (https://ijgvd.megabank.tohoku.ac.jp, [17].

### Plasmids

The Myc-DDK-tagged pCMV-PAX9 expression vector (RC200380) was purchased from OriGene Technologies (Rockville, MD, USA) and named pAM6. P20L mutation was introduced by replacing the 0.3-kb EcoRI-BspEI fragment of pAM6 with a synthesized fragment containing the mutation (GeneArt Gene Synthesis Service, ThermoFisher Scientific). A240P mutation was introduced by PCR-based site-directed mutagenesis using a pair of primers. For the null mutant, a 0.3-kb BglII-BspEI fragment containing the first ATG was deleted from pAM6. These plasmids were named pAM7 (P20L), pAM8 (A240P), pAM9 (P20L/A240P) and pAM4 (null). A reporter plasmid was constructed by replacing the SV40 early promoter of the pGL4.13 luciferase reporter vector (Promega, Fitchburg, WI, USA) with a 2.3-kb BMP4 promoter fragment and named pAM5. Nucleotide sequences were verified after the construction.

### Cultured cells

COS-7 cells were kindly provided by Prof. K. Furuyama at Iwate Medical University in February 2016[18] and cultured in DMEM (Thermo Fisher Scientific) supplemented with 10% fetal bovine serum in a humidified 5% CO_2_ atmosphere at 37°C.

### Luciferase reporter assay

One million COS-7 cells per well were seeded into 6-well plates and cultured for 24 h. A set of the reporter plasmid pAM5 (0.4 μg) and either one of the PAX9 expression plasmid (pAM4, 6, 7, 8, or 9; 2 μg) and the calibration plasmid pRL-SV40 (Promega; 0.1 μg) was co-transfected using Lipofectamine® 3000 Reagent (Thermo Fisher Scientific) in accordance with the manufacturer’s instructions. The cells were harvested at 24 h after transfection and split into 96-well plates with a white bottom (4×10^4^ cells/well) for luciferase assay and 6-well plates (1×10^6^ cells /well) for protein preparation (see below). At 48 h after transfection, the cells were tested for luciferase activity with a Dual-Glo Luciferase Assay System (Promega) and Centro LB 960 Microplate Luminometer (Berthold Technologies, Bad Wildbad, Germany). The reporter assay experiment was repeated at least 3 times with independent transfection. Fitting to a linear model was done to evaluate the effect of P20L and A240P on luciferase activity using R [19] on Rstudio Desktop (Rstudio Inc., Boston, MA, USA).

### Protein preparation and immunoblot analysis

At 48 h after transfection, the cells were washed twice with ice-chilled 0.15 M NaCl, fixed with 10% TCA in 0.15 M NaCl at 4°C overnight, and then scraped off into a tube. The cell pellet was washed twice with ice-chilled deionized water and lysed in 9 M urea, 2% Triton X-100 and 1% DTT. Protein concentration was measured with a BCA protein assay kit (Merck Millipore Corporation, Billerica, MA, USA) before addition of DTT. Approximately 30 μg of protein per lane was electrophoresed on 10% SDS-PAGE gel for 45 min at 200 V and then transferred onto FluoroTrans® W polyvinylidene fluoride membranes (Pall Corporation, Port Washington, NY, USA). The membranes were blocked with 5% non-fat dried milk (Cell Signaling Technology, Danvers, MA, USA) in 1×TBS-T for 1 h at room temperature and then immunoreacted with mouse monoclonal anti-Myc antibody (sc-40, Santa Cruz Biotechnology, Dallas, TX, USA) or rabbit monoclonal anti-GAPDH antibody (#5174S, Cell Signaling Technology) at 1:1000 dilution overnight at 4°C. After two washes with 1×TBS-T, the membranes were reacted with HRP-conjugated secondary antibodies (GE Healthcare Life Sciences, Chicago, IL, USA) at 1:4000 dilution for 1 h at room temperature. Signals were visualized with ECL Prime Western Blotting Detection Reagent (GE Healthcare Life Sciences) and ChemiDoc XRS (Bio-Rad Laboratories, Hercules, CA, USA). Intensity of the signals was quantified using ImageJ/Fiji software [20].

### Electrophoretic mobility shift assay

EMSA was performed as described previously [21,22] using a LightShift™ Chemiluminescent EMSA Kit (Thermo Fisher Scientific). A nuclear extract was prepared using NE-PER™ Nuclear and Cytoplasmic Extraction Reagents (Thermo Fisher Scientific) at 48 h after transfection of one million COS-7 cells with 2.5 μg of pAM6, pAM7 or pAM4. Expression of PAX9-Myc and integrity of nuclear extract were confirmed by immunoblotting with anti-Myc antibody or anti-Lamin B antibody (sc-6217, Santa Cruz Biotechnology). A pair of 5'-biotinylated oligonucleotides, CD19-2(A-ins) (5'-CGTGGTCACGCCTCAGTGCCCCA-3') and its antisense (5'-TGGGGCACTGAGGCGTGACCACG-3') was annealed and then incubated with the nuclear extract at room temperature for 30 min. For specific competition for binding, a 200-fold excess of non-biotinylated sequences was included in the reaction. For super-shift of the PAX9-bound complex, anti-Myc antibody was added. The entire reaction mixture was run on a non-denaturing 0.25×TBE 6% polyacrylamide gel for 30 min at 200 V at 4°C and then transferred onto Biodyne® B nylon membranes (Pall Corporation). Signals were visualized with reagents included in the kit and ChemiDoc XRS (Bio-Rad Laboratories).

### Modeling of the mutant PAX9 paired domain structure

The three-dimensional structure of the wild-type PAX9 paired domain was inferred using Swiss-model (https://www.swissmodel.expasy.org) with PDB:6PAX as a template. The model was recombined with DNA chains in 6PAX by Pymol (https://www.pymol.org) and the resulting PDB file was optimized with RepairPDB of FoldX (http://foldxsuite.crg.eu). The structure of the P20L variant was modeled using BuiltModel of FoldX. The stability of the PAX9-DNA complex was calculated with AnalyseComplex with the complexWithDNA option true.

## Results

### Confirmation of the non-syndromic status of tooth agenesis by clinical examination

**Fig 1:**
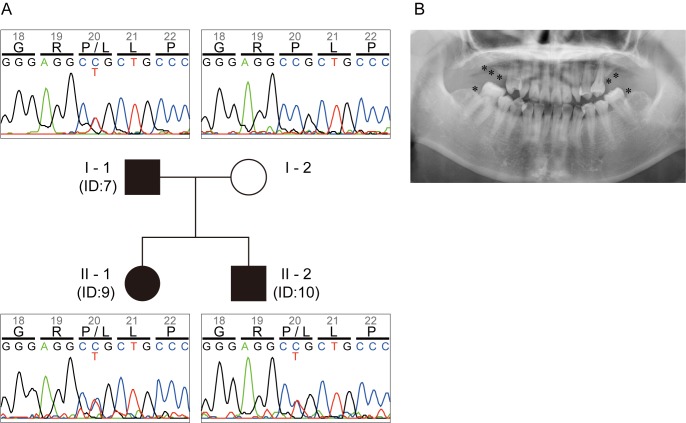
Pedigree with tooth genesis involving three patients. (A) Proband (patient ID 9 in Table 1), her father (ID 7) and brother (ID 10) are affected (indicated with filled symbols), while her mother is unaffected (indicated with an open symbol). The inheritance pattern is consistent with autosomal dominance. The three affected members invariably show C/T heterozygosity at the second position of the Pro^20^ codon, while the unaffected member shows C/C homozygosity. (B) Radiogram of the proband (patient ID 9 in Table 1). Missing teeth other than 3rd molars are indicated with asterisks.

None of the nine patients exhibited significant health problems other than selective tooth agenesis and associated microdontia. The number of missing teeth ranged from 1 to 9 with a mean of 5.2 (Table 1). In the familial case involving the three patients from two generations (IDs 7, 9 and 10), the missing teeth were predominantly first and second molars (Table 1, Fig 1B), which is a recurrent pattern noted in *PAX9*-mutated cases [23]. Inheritance of the phenotype was consistent with Mendelian dominance of a single responsible variant (Fig 1A). For the six sporadic cases, defects in premolars were most common, whereas those in lateral incisors or canines were also observed (Table 1). Clinical interview of the patients in the sporadic cases did not reveal any family history of tooth agenesis, suggesting an inheritance other than Mendelian dominance, or involvement of a *de novo* germline mutation.

**Table 1:** Summary of the patients investigated in the present study.

ID	Gender	Age	# missing teeth*		Right								Left							
					8	7	6	5	4	3	2	1	1	2	3	4	5	6	7	8
7	M	57	2	Upper	●	○				●	○				○				○	●
				Lower	●														●	●
9	F	28	7	Upper	●	●	●	●		○					○			●	●	●
				Lower	●	●													●	●
10	M	26	8	Upper	●	●	●	●			○						●	●	●	●
				Lower	●	●													●	●
13	F	33	9	Upper				●	●	●	●			●	●	●	●			
				Lower							●									
16	F	24	4	Upper				●									●			
				Lower				●									●			
23	F	18	4	Upper																
				Lower				●			●			●			●			
25	F	15	6	Upper				●									●			
				Lower				●	●							●	●			
26	F	22	6	Upper						●	●				●		●			
				Lower						●					●					
27	F	28	3	Upper				●			○									
				Lower				●									●			

●: Tooth agenesis

○: Microdens

*: excluding 3rd molars.

### A P20L variant of PAX9 revealed by mutation analysis

By sequencing of the *PAX9* coding regions in all nine patients, we identified a transition mutation, NM_006194.3:c.59C>T, that results in a proline to leucine amino acid substitution, P20L. Although identical mutation was previously reported in an Indian family with tooth agenesis, since experimental evidences for its causality is lacking [15,16], we decided to investigate this mutation in detail. The variant allele was shared exclusively by the three familial patients as heterozygotes, and not by an unaffected member of the family (Fig 1A). All of the familial patients possessed another missense mutation, A240P, in *PAX9* as homozygotes. This allele, however, was also observed in the unaffected member and found to be a common variant (rs4904210, allele frequency is 0.4653 in Integrative Japanese Genome Variation Database), therefore it was considered unlikely to be responsible for tooth agenesis. In the six sporadic cases, no non-synonymous variants other than PAX9 A240P and MSX1 A40G (rs36059701, allele frequency 0.0760) were identified in the coding regions of *PAX9* and *MSX1*.

**Fig 2:**
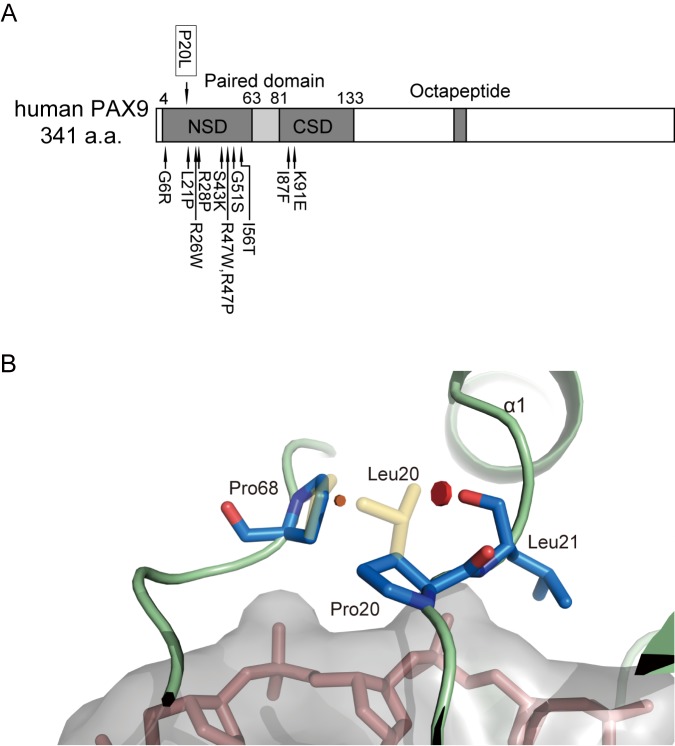
Position of the identified PAX9 mutation. (A) Locations of P20L and previously reported missense variants are shown with arrows above and below the PAX9 diagram, respectively [12,25–31]. Reported pathogenic variants with nonsense mutations or frameshifts are omitted. NSD, N-terminal subdomain of the paired domain; CSD, C-terminal subdomain of the paired domain. (B) Modeled pair-domain structure of the wild-type (blue and red with green backbone) and P20L (yellow) PAX9. P20L mutation results in Van del Waals clashes with the carbonyl oxygen of Leu^21^ (red disc) and the side chain of Pro^68^ (brown disc). This results in a slight displacement of Pro^68^. DNA is shown in pink with a gray surface.

The Pro^20^ resides in the loop between the β hairpin and the α1 helix within the N-terminal subdomain (NSD) of the paired domain, and is invariant among nine PAX family proteins in humans (Fig 2A). Notably, a proline to leucine substitution at the homologous site of PAX3 has been reported to be responsible for a case of Waardenburg syndrome (P50L, rs104893650, [24]. PolyPhen-2 analysis predicted that this PAX9 variant would be "probably damaging" (the score being 1.0, the highest possible). We then constructed homology models of the WT and P20L PAX9 paired domain with Swiss-model and FoldX. The models showed that P20L may introduce a Van der Waals clash with the backbone carbonyl group of Leu^21^ and the sidechain of Pro^68^ (Fig 2B), although not apparently creating an obvious structural change in the DNA interface. AnalyseComplex of FoldX predicted that the P20L mutation might decrease the interaction energy with DNA (ΔΔG = 1.0 kcal/mol) and impose an additional cost in terms of intramolecular Van der Waals clashes (ΔΔG = 1.9 kcal/mol) compared with the wild-type PAX9. AnalyseComplex against R26W variants for comparison resulted in a 1.3 kcal/mol decrease of interaction energy and an additional 4.9 kcal/mol Van der Waals clash. Taken together, the data suggest that the P20L mutation is likely to result in mitigation of PAX9 function, whereas its effect could be milder than R26W.

### Reporter assay verification of the impact of P20L mutation on PAX9 function

**Fig 3:**
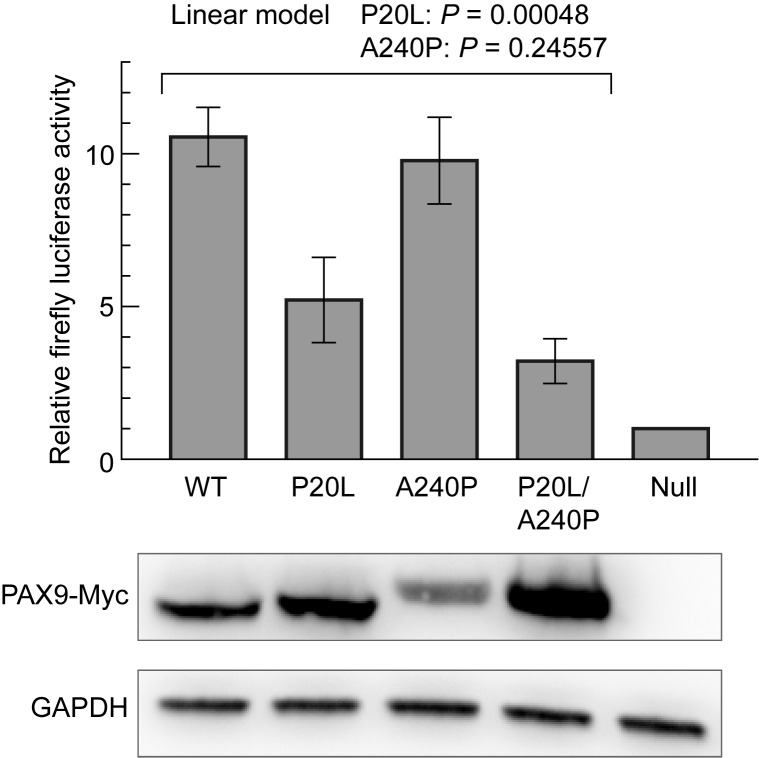
Luciferase reporter assay using the *BMP4* promoter region as a cis element. Activity of firefly luciferase expressed from the reporter vector was normalized with *Renilla* luciferase, and shown as arbitrary units (mean ± SEM). Experiments were repeated three times with independent transfections, and for each experiment luciferase activity was measured in triplicate. Protein samples were prepared from aliquots of transfected cells, and ectopic PAX9 expression was verified by immunoblotting. An alleviating effect of P20L on transactivation was supported statistically, while that of A240P was not, as shown above the graph.

To experimentally assess how P20L mutation affects PAX9 function as a transcription factor, we ectopically expressed the wild-type or variant PAX9s in COS-7 cells and measured their activity by reporter assay using the 2.3-kb promoter region of *BMP4* as a cis element, an established target of PAX9 [32]. We noticed that *PAX9* transgenes harboring P20L mutation (P20L and P20L/A240P in Fig 3 bottom) reproducibly attained a higher expression of its own than those not harboring P20L (wild-type and A240P, 2.5 fold (geometric mean), see S1 Table). In spite of this, P20L or P20L/A240P PAX9s exhibited much lower activity in transactivation of luciferase expression than the wild-type or A240P PAX9 (Fig 3 top and S2 Table). Fitting of relative luciferase activities to a linear model showed that the P20L variant significantly attenuated the PAX9 activity while A240P did not. However, P20L did not completely abolish the transactivation of PAX9, and some residual activity was observed relative to the null allele plasmid.

### P20L mutation compromises DNA binding activity

**Fig 4:**
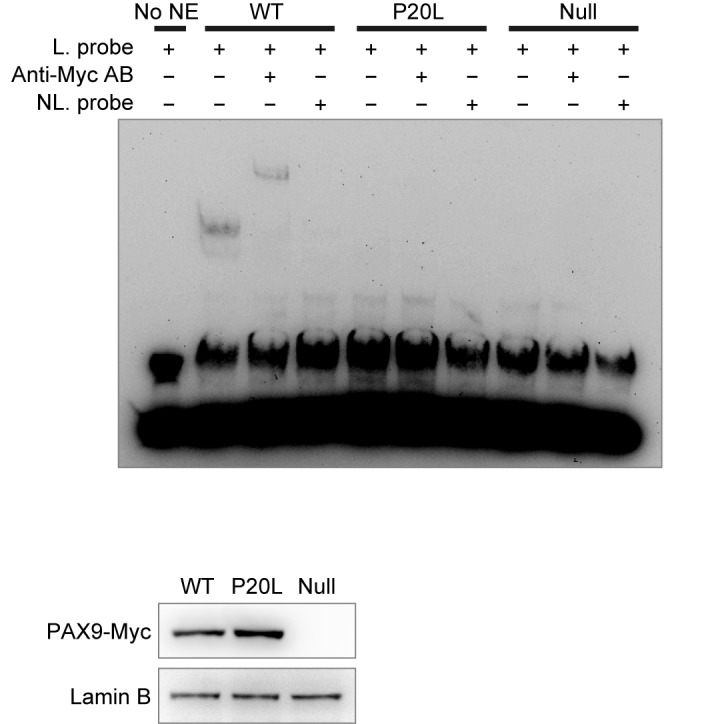
Electrophoretic mobility shift assay of PAX9^WT^ and PAX9^P20L^. Nuclear extract was prepared at 48 h after transfection with PAX9-Myc-expressing (WT or P20L) or 1st ATG-deleted plasmids (Null), and analyzed using the CD19-2(A-ins) probe. Ectopic expression of PAX9-Myc and integrity of nuclear extract were confirmed with immunoblotting (bottom). Excessive amount of the non-biotinylated DNA eliminated the mobility shift signals in PAX9^WT^, whereas addition of anti-Myc antibody resulted in a super-shift, confirming the specificity of the bipartite interaction. No detectable degree of signal shift was observed in PAX9^P20L^. No NE, no nuclear extract added; L probe, biotin-labelled probe; NL probe, non-labelled probe.

To evaluate the effect of P20L mutation on the specific DNA binding activity of PAX9, we performed an electrophoretic mobility shift assay using the CD19-2(A-ins) sequence, a consensus for paired domain binding, as a probe [21]. The nuclear extract prepared from COS-7 cells expressing wild-type PAX9-Myc caused a clear shift in the mobility of the probe, which was super-shifted with anti-Myc antibody and titrated out with an excess amount of non-labeled competitor DNA (Fig 4). In contrast, the nuclear extract from cells expressing the P20L mutant showed virtually no sign of specific binding under the conditions we employed.

## Discussion

The majority of pathogenic missense mutations of PAX9, including P20L investigated in the present study, reside in NSD of the paired domain. In addition to the higher degree of NSD conservation among PAX proteins, the ratio of missense variants to synonymous variants is significantly lower in NSD of PAX9 (4 to 14) than in CSD (16 to 6, *P* = 0.00364 by Fisher’s exact test) in the ExAC database. This suggests that NSD may be under stronger purifying selection in the human population, and therefore functionally and/or structurally more crucial. A similar pattern of pathogenic mutation distribution is more clearly represented in another PAX family member, PAX3 [33]. How this reconciles with the proposed role of CSD in specific DNA binding is currently unclear [34]. A further search for pathogenic variants or large-scale mutagenesis experiments using an appropriate assay system will be required to address this question.

Since highly conserved Pro^20^ is located at the protein-DNA interface, its substitution would be naturally implicated in any possible defect of DNA interaction. Our present study showed that DNA binding activity as well as transactivation activity of the variant PAX9 was severely compromised. On the other hand, homology modeling of PAX9 P20L did not pick up large structural anomalies in the DNA interface other than slight displacement of a few nearby amino acid residues. Whereas this may likely be due to modeling limitation, it also might be connected to the relatively mild clinical phenotype of P20L (in the present study, the mean number of missing teeth was 5.7) compared with other pathogenic variants such as R26W (11.3) [26]. In fact, foldX analysis predicted that R26W might impose a markedly higher cost of intramolecular Van der Waals clashes than P20L. The apparent residual activity of P20L variants in the reporter assay supports this notion.

Intriguingly, when ectopically expressed in cultured COS-7 cells, P20L protein reproducibly attained a higher expression level than its wild-type counterpart. Our preliminary study did not detect clear difference in stability between the wild-type and the P20L mutant proteins. Although we have no evidence that this takes place *in vivo*, higher expression of the mutant protein might have some effect on the resulting phenotype besides the defect in biochemical activity. This possibility needs to be explored in a future study.

We were not successful in identifying candidate mutations in *PAX9* and *MSX1* in the six sporadic cases in the present study. Since previous studies involving large numbers of subjects have found that the incidence of phenotypes attributable to PAX9 or MSX1 mutation is in the range of several percent [31,35], this was not unexpected. Those studies found a surprisingly high frequency of *WNT10A* mutation in patients with tooth agenesis. This could be the locus of mutations that were responsible for some of the sporadic cases in the present study, as well as other reported loci such as *AXIN2* or *EDA*.

It has been well documented that the pattern of tooth absence in congenital tooth agenesis is associated with causative loci and variants [23]. Knowledge of their interrelationship would be helpful for identifying the genetic alterations responsible, and this would enable early clinical intervention. A previous study using a mouse model has suggested that for some causative mutations appropriate intervention could permanently correct the tooth phenotype [36]. Such information together with the mechanistic background should be accumulated through future research.

## Supporting information

S1 Fig Uncropped WB for PAX9-Myc (corresponding to Fig 3). (TIF)

S2 Fig Uncropped WB for PAX9-myc (size marker, corresponding to Fig 3). (TIF)

S3 Fig Uncropped WB for GAPDH (corresponding to Fig 3). (TIF)

S4 Fig Uncropped EMSA gel (corresponding to Fig 4). (TIF)

S5 Fig Uncropped WB for PAX9-Myc (corresponding to Fig 4). (TIF)

S6 Fig Uncropped WB for PAX9-Myc (size marker, corresponding to Fig 4). (TIF)

S7 Fig Uncropped WB for Lamin B (corresponding to Fig 4). (TIF)

S8 Fig Uncropped WB for Lamin B (size marker, corresponding to Fig 4). (TIF)

S1 Table Expression level of PAX9-Myc in the reporter assay. (XLSX)

S2 Table Primary data for the reporter assay (corresponding to Fig 3). (XLSX)
