# Supplementary figures and images for: Characterization of PAX9 variant P20L identified in a Japanese family with tooth agenesis
Source: PLoS One. 2017 Oct 12;12(10):e0186260. doi: 10.1371/journal.pone.0186260 (PMC5638407; doi:10.1371/journal.pone.0186260)

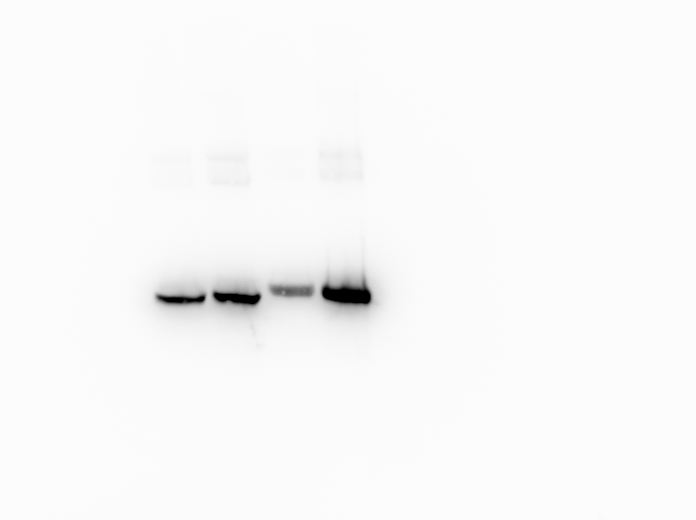

Supplement: S1 Fig — (TIF) [file pone.0186260.s001.tif]

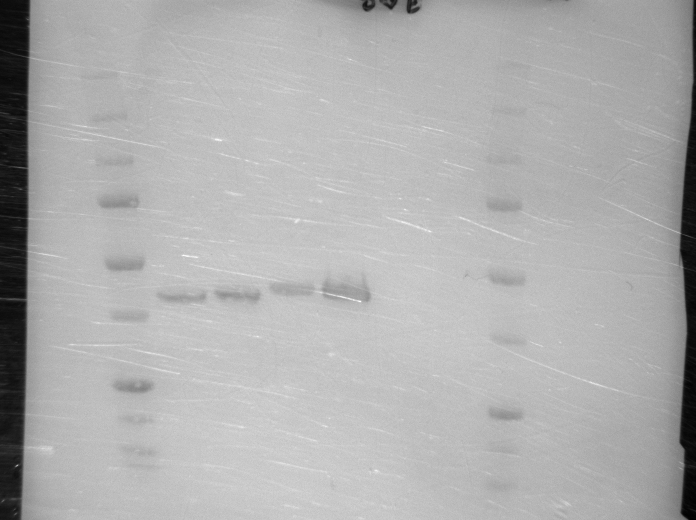

Supplement: S2 Fig — (TIF) [file pone.0186260.s002.tif]

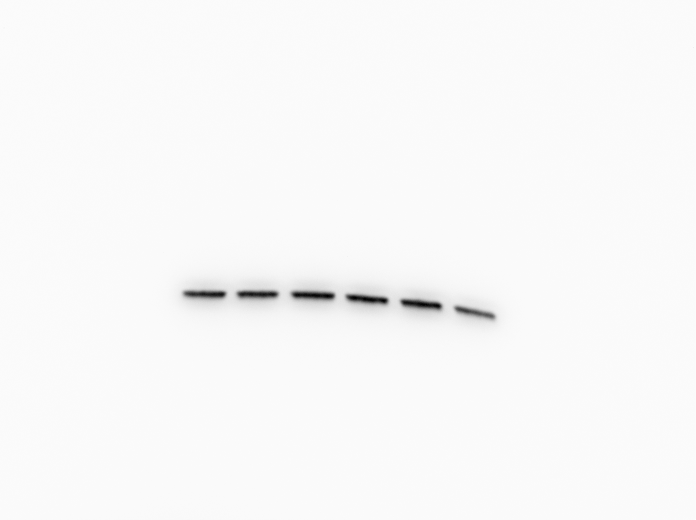

Supplement: S3 Fig — (TIF) [file pone.0186260.s003.tif]

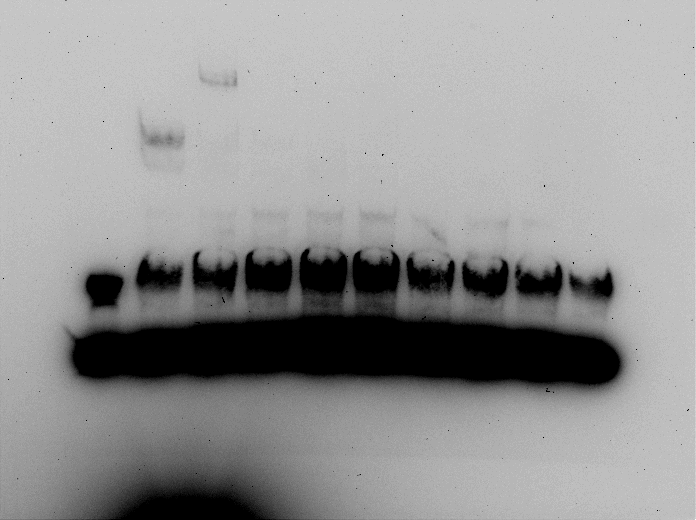

Supplement: S4 Fig — (TIF) [file pone.0186260.s004.tif]

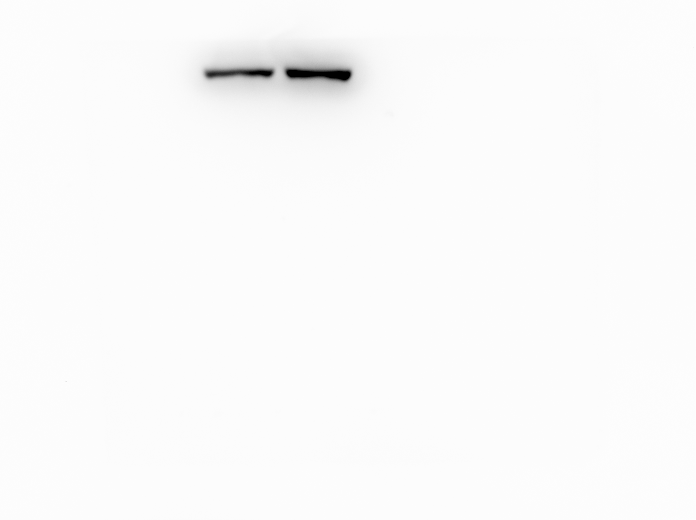

Supplement: S5 Fig — (TIF) [file pone.0186260.s005.tif]

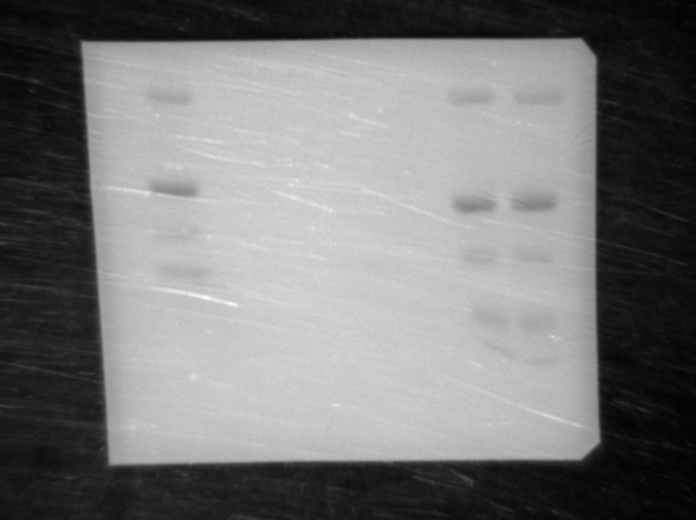

Supplement: S6 Fig — (TIF) [file pone.0186260.s006.tif]

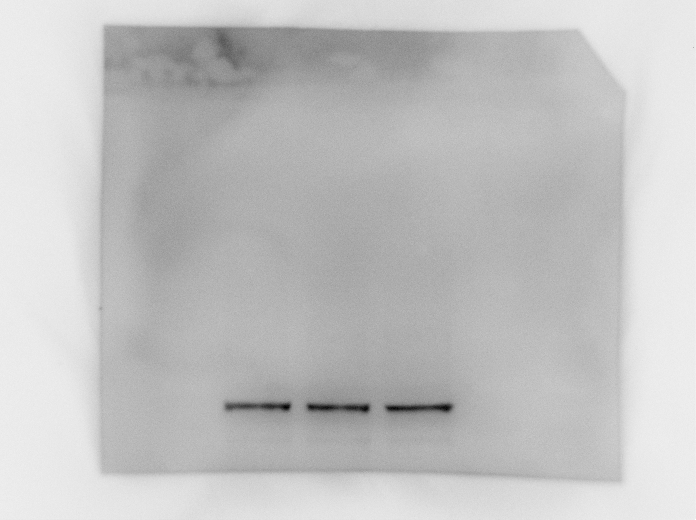

Supplement: S7 Fig — (TIF) [file pone.0186260.s007.tif]

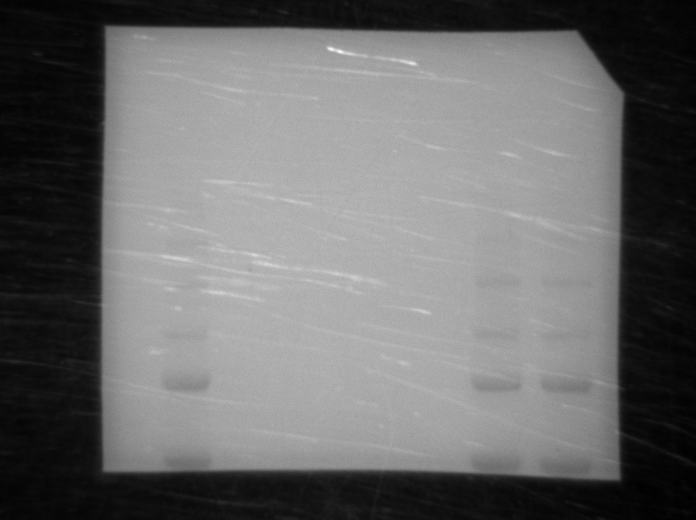

Supplement: S8 Fig — (TIF) [file pone.0186260.s008.tif]
